# Rare Copy Number Variants in Isolated Sporadic and Syndromic Atrioventricular Septal Defects

**DOI:** 10.1002/ajmg.a.35315

**Published:** 2012-04-23

**Authors:** James R Priest, Santhosh Girirajan, Tiffany H Vu, Aaron Olson, Evan E Eichler, Michael A Portman

**Affiliations:** 1 Department of PediatricsUniversity of Washington School of Medicine, Seattle, Washington; 2 Seattle Children’s Hospital Research InstituteSeattle, Washington; 3 Department of Genome Sciences, Howard Hughes Medical InstituteUniversity of Washington School of Medicine, Seattle, Washington; 4 University of Washington School of MedicineSeattle, Washington; 5 Division of Cardiology, Department of PediatricsUniversity of Washington School of Medicine, Seattle, Washington

**Keywords:** Down syndrome, atrioventricular septal defects, copy number variation, array CGH, congenital heart disease

## Abstract

Atrioventricular septal defects (AVSDs) are a frequent but not universal component of Down syndrome (DS), while AVSDs in otherwise normal individuals have no well-defined genetic basis. The contribution of copy number variation (CNV) to specific congenital heart disease (CHD) phenotypes including AVSD is unknown. We hypothesized that de novo CNVs on chromosome 21 might cause isolated sporadic AVSDs, and separately that CNVs throughout the genome might constitute an additional genetic risk factor for AVSD in patients with DS. We utilized a custom oligonucleotide arrays targeted to CNV hotspots that are flanked by large duplicated segments of high sequence identity. We assayed 29 euploid and 50 DS individuals with AVSD, and compared to general population controls. In patients with isolated-sporadic AVSD we identified two large unique deletions outside of chromosome 21 not seen in the expanded set of 8,635 controls, each overlapping with larger deletions associated with similar CHD reported in the DECIPHER database. There was a small duplication in one patient with DS and AVSD. We conclude that isolated sporadic AVSDs may be occasionally associated with large de novo genomic structural variation outside of chromosome 21. The absence of CNVs on chromosome 21 in patients with isolated sporadic AVSD suggests that sub-chromosomal duplications or deletions of greater than 150 kbp on chromosome 21 do not cause sporadic AVSDs. Large CNVs do not appear to be an additive risk factor for AVSD in the DS population. © 2012 Wiley Periodicals, Inc.

## INTRODUCTION

Approximately 75% of congenital heart disease (CHD) is sporadic, occurring separately from conditions such as Down syndrome (DS) or 22q11.2 deletion syndrome suggesting that genetic basis for the majority of CHD remains clinically unrecognized Goldmuntz and Lin, 2008. Atrioventricular septal defects (AVSD) comprise a spectrum of congenital heart malformations showing deficiencies in the structures that normally separate the atrial and ventricular chambers. Without an associated syndrome, AVSDs occur at a rate of 0.98–1.32 per 10,000 births Harris et al., 2003. In striking contrast, approximately 17% of individuals with DS manifest with AVSD, and nearly 50% of fetuses diagnosed prenatally with an AVSD displayed a major chromosomal abnormality Huggon et al., 2000; Freeman et al., 2008. Significant efforts have sought to delineate a phenotype-specific interval on chromosome 21 that confers the 3,000-fold increase in relative risk for AVSDs in individuals with DS compared to the general population. While studies of subchromosomal duplications of chromosome 21 suggest 21q22.3 as the causative interval Korenberg et al., 1992; Davies et al., 1993; Sinet et al., 1994; Korbel et al., 2009, linkage studies of familial non-syndromic AVSDs from the pre-genomic era have not found a causative interval on chromosome-21 shared with the DS population, or within the remainder of the genome Wilson et al., 1993; Cousineau et al., 1994; Digilio et al., 1999. Other studies of AVSD associated 3p- syndrome and recurrent 8p23.1 deletions, demonstrate that there are likely multiple AVSD risk loci with incomplete penetrance scattered throughout the genome, and that genomic structural variants such as copy number variations (CNVs) are potentially etiologic in the pathogenesis of CHD Shuib et al., 2009; Wat et al., 2009.

We had two objectives for this study examining the relationship of CNVs and AVSDs: (1) to survey the whole genome for large CNVs to determine if variants on Chromosome 21 or other syndromic-AVSD loci were associated with isolated sporadic AVSDs; and (2) to determine if sporadic CNVs play an additive role in the pathogenesis of AVSD in patients with DS.

## MATERIALS AND METHODS

### Patient Ascertainment

The study was approved by the institutional review board of Seattle Children’s Hospital, and informed consent was obtained for each DNA sample obtained. We initially identified patients with an AVSD from the electronic medical record, and excluded those with congenital malformations, developmental delay, or other major CHD. Patients with DS were included but those with a family history of CHD, DS, or other congenital malformation were excluded if reported by the parents or if discovered in the electronic medical record. The presence of an AVSD with situs solitus was confirmed from echocardiogram or operative report. All non-syndromic patients were clinically assessed by pediatric cardiologists and/or geneticists to exclude other syndromes associated with AVSD. A clinical report of a diagnostic karyotype was criteria for enrollment for patients with DS, but not for patients with isolated sporadic AVSD. When available and willing, interested parents were also invited to participate though parent participation was not criteria for enrollment of subjects with AVSD.

### CNV Detection

DNA was isolated by standard techniques from whole blood samples from patients and saliva from parents. To assay for CNVs, we used a genomic hotspot array containing a total of 135,000 probes (Roche NimbleGen, Madison, WI). The hotspot chip consists of a high density of probes (one probe every 2.6 kbp) in regions flanked by segmental duplications Bailey et al., 2002 and a lower probe density (one probe every 36 kbp) in the genomic backbone. All experiments were performed as described previously using a male reference DNA (GM15724) obtained from Coriell Laboratories (Camden, NJ). Following hybridization we calculated means and standard deviations for each chromosome using the observed log intensity ratios and then performed z-score transformations. A Hidden Markov Model (HMM) was employed to bin probe log intensity ratios and sort binned probes into increased, normal, or decreased copy number. Given the accuracy of our platform, we restricted our analysis to bins of 10 or more probes spanning 150 kb or larger. Discovered CNVs were assessed for inheritance in parental DNA samples when available and confirmed on a second high-density 400,000-probe Agilent chip of similar design with density probe density of 850 bp in the genomic hotspots and 14 kbp in the genomic backbone [Girirajan and Eichler, unpublished]. For the Agilent arrays, data analysis was performed following feature extraction using DNA analytics with ADM-2 setting and the Genomic Workbench software according to manufacturer’s instructions. Finally, CNVs were compared to databases of known structural variation and fine scale mapping of 8,329 healthy adults Cooper et al., 2011 and 306 NIMH control samples analyzed on the same microarray platform Vu et al., 2011. All signal intensities were uploaded on to a custom UCSC genome browser (Build 36) for visual inspection.

## RESULTS

We enrolled 79 patients with AVSDs, 29 euploid patients, and 50 DS patients all without other congenital malformations, other CHD, or major pathology (Supplementary eTable I—see Supporting Information online). The enrolled patients with isolated sporadic AVSD were without developmental delay. To ensure the validity of CNV calls we used previously derived post hoc quality controls Vu et al., 2011 of an absolute *z*-score > 1.5. After filtering for the described criteria and excluding the sex chromosomes we obtained 463 CNVs. We eliminated common copy number polymorphisms and CNVs with a reciprocal overlap of 50% or more of their length to those in a total of 8,635 normal individuals Cooper et al., 2011; Vu et al., 2011. After filtering for common copy number polymorphisms and variants identified at an allele frequency of >0.1% (>9/8,329 controls) in the control population, we identified a total of three rare CNVs. None of these CNVs were observed in our total expanded set of 8,635 control individuals analyzed on the same platform or curated from various SNP array data on individuals with no structural cardiac phenotypes.

**FIG 1:**
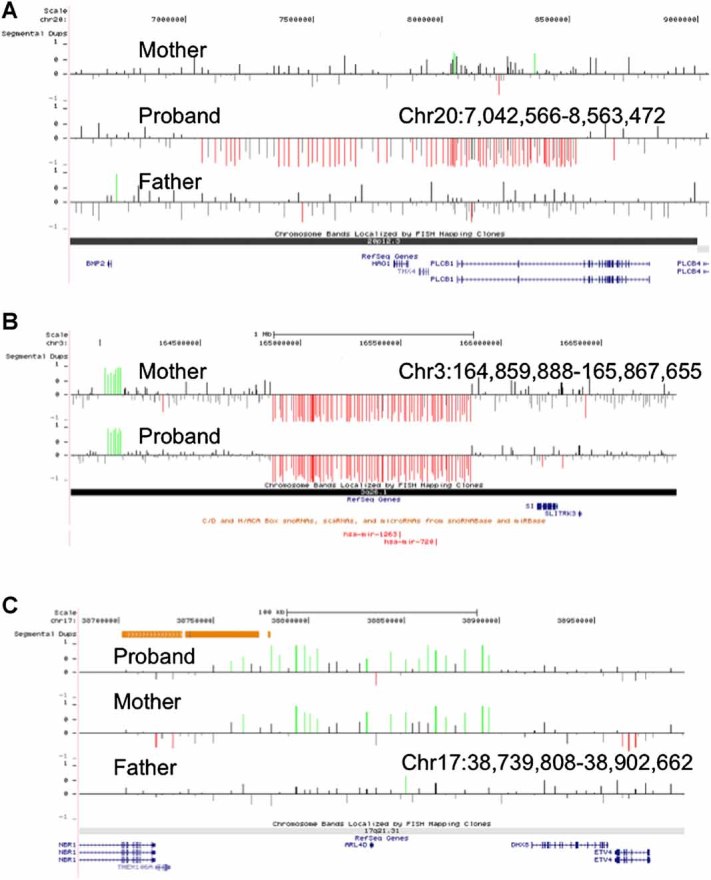
Rare CNVs in AVSD with and without DS. A: This euploid patient exhibited a large de novo deletion of 1.5 Mbp at 20p12.3 containing phospholipase C beta subunit *PLCB1* which is a component of a cardiac sarcolemma associated G protein signaling complex regulating cardiomyocyte hypertrophy. B: A second euploid patient had a 1 Mbp deletion at 3q26.1 without any transcribed genes but containing enhancers of heart expression and *hsa-miRNA-1263* which regulates a number of genes related to cardiac development and CHD including *CHD7*, *SMAD7*, and *MID1* (Supplementary eTable II—see Supporting Information online). C: This DS patient had a small 163 kbp duplication at 17q21.31 overlapping an ADP ribosylation factor *ARL4D* which has no known cardiac function.

Within the 29 genotyped patients with isolated sporadic AVSD, two individuals (6.8%) had readily identifiable unique CNVs. One male with a complete AVSD had a 1 Mbp deletion at 3q26.1 inherited from an unaffected mother containing no protein coding genes but two micro-RNA genes (Fig. 1). A second male with a partial AVSD exhibited a large de novo deletion of 1.5 Mbp at 20p12.3 containing three protein coding genes; *HAO1*, *TMX4*, *PLCB1*. Only one of 50 genotyped DS patients had a small 163 kbp duplication at 17q21.31 of inherited from an unaffected mother containing one protein coding gene *ARL4D* (Table I).

**TABLE I:** Rare Copy Number Variants in AVSD

Location (hg18)	Locus	Size (kbp)	Defect	AVSD phenotype	Involved genes	Comments	DECIPHER correlates
EUPLOID with AVSD							
Chr20:7,042,566–8,563,472	20p12.3	1,521	Deletion	Partial	*HAO1*, *TMX4*, *PLCB1*	De novo event. PLCB1 is a regulator of cardiomyocyte hypertrophy	Patient 1,578 with 19.1 Mb deletion at 20p13–20p11.23 with ASD, VSD, and other congenital anomalies
							Patient 1,692 with a 3.4 Mb deletion at 20p12.2–20p12.1 with an ASD
Chr3:164,859,888–165,867,655	3q26.1	1,008	Deletion	Complete	*None*	Inherited from an unaffected mother. Contains *hsa-mIR-1263* which may regulate a variety of genes related to cardiac development, and *hsa-mIR-720* which is expressed in the heart	Patient 250,665 with 29.9 Mb deletion at 3q22–3q26 with multiple congenital anomalies including VSD
TRISOMY 21 with AVSD							
Chr17:38,739,808–38,902,662	17p21.31	163	Duplication	Complete ECD	*ARL4D*	Inherited from an unaffected mother. Adjacent to *NBR1* a ubiquitin protease regulator of fetal cardiomyocyte hypertrophy. *ARL4D* has no known cardiac function	None

## DISCUSSION

We hypothesized that CNVs on chromosome 21 might be associated with AVSD in otherwise normal individuals. To this end we evaluated patients with isolated sporadic AVSD without DS for CNVs in order to identify novel potential candidate genes for this cardiac malformation. Within the cohort of isolated sporadic AVSD we identified two unique sub-chromosomal deletions associated with AVSD. Neither of these discovered variants occurred on chromosome 21, but instead each of the deletions involves discreet genomic regions that previously have not been directly linked to AVSD. The absence of de novo CNVs on chromosome 21 is consistent with prior linkage based studies of familial AVSD which excluded chromosome 21 in the pathogenesis of isolated sporadic AVSD without identifying other loci within the genome Wilson et al., 1993; Cousineau et al., 1994; Digilio et al., 1999. We also hypothesized that de novo CNVs might contribute to the development of AVSDs in patients with DS, but found only one additional lesion within this population inherited from an unaffected parent.

Despite the absence of CNVs discovered on chromosome 21 in a population with isolated sporadic AVSD, at least one of the deletions can plausibly offer a causative mechanism in that individual patient. The de novo 1.5 Mb deletion at 20p12.3 covers *PLCB1* which encodes a phospholipase C beta subunit. Interestingly, *PLCB1* is a component of a cardiac sarcolemma associated G protein signaling complex that inhibits G protein mediated cardiomyocyte hypertrophy in vitro Filtz et al., 2009; Grubb et al., 2011. Further circumstantial evidence is provided by the DECIPHER database, which reports two patients with multiple congenital defects including both ASD and VSD with large deletions overlapping *PLCB1* (Table I) Firth et al., 2009.

The deletion found at 3q26.1 in a euploid male with complete AVSD was inherited from an unaffected mother, which weakens our hypothesis of causality. Though this 1 Mb deletion does not contain any identified genes, the region encompasses four predicted enhancers of heart specific transcription, *hsa-miR1263* a microRNA that has a variety of predicted targets also related to cardiac development (Supplemental eTable II—see Supporting Information online), and *hsa-mIR-720* which is known to be expressed in the heart Tang et al., 2007; Friedman et al., 2009; Narlikar et al., 2010; Zhang et al., 2011. Our 1 Mb deletion also overlaps with a 29.9 Mb deletion reported in the DECIPHER database in a patient with a VSD among many other congenital defects.

Within one patient with DS, a163 kbp duplication at 17q21.31 also inherited from an unaffected mother overlaps an ADP ribosylation factor *ARL4D* that has no experimentally described cardiac function, a microRNA without known targets, and a number of uncharacterized transcripts. Furthermore there are no patients in the DECIPHER with overlapping genetic lesions and CHD. Additional data are necessary to support that this particular duplication confers additional risk for AVSD. Our study design did not include genotyping of control patients with DS who were free of CHD. Thus the absence of discovered CNVs within the DS population does not exclude the possibility of CNVs protective against AVSD or CHD within the DS population.

As we have provided no direct experimental evidence of causality, either the 3q26.1 or 20p12.3 deletions could indeed be unrelated to the cardiac phenotype. Alternatively, either CNV might confer a significant risk for AVSD with incomplete expressivity, similar to Trisomy 21. Our study was limited to only one type of genetic variation as we did not genotype for individual nucleotide differences. Additionally, the design of our Nimblegen screening platform allowed us only to confidently genotype CNVs greater than 150 kbp, thus the contribution of smaller variants cannot accurately be excluded. Other important limitations to our study include a small cohort that limited our power to detect genetic differences occurring in multiple individuals. Additionally, in the isolated sporadic AVSD population we cannot completely exclude a clinically subtle presentation of Ellis-van Creveld, heterotaxy, Holt-Oram, or other AVSD associated syndromes. Likewise, our methodology for genotyping and analysis was not designed to assess the impact of common copy number polymorphisms upon the development of AVSD phenotype Campbell et al., 2011.

The study of the genetic basis of CHD is complicated by the significant phenotypic as well as genetic heterogeneity evident with both defined genetic lesions and defined CHD phenotypes. While multiple genetic loci have been associated with a specific structural cardiac defect such as ToF, the same genetic lesion may also be associated with multiple types of CHD. For example, the 22q11.2 deletion that is classically associated with ToF is also frequently found in patients with interrupted aortic arch, isolated ventricular septal defects, truncus arteriosus, and various other type of CHD Botto et al., 2003. A recent study crossing *NKX-2.5* knockout mice onto different genetic backgrounds has elegantly demonstrated that multiple genetic loci influence both the incidence of CHD and the type of CHD expressed Winston et al., 2010.

Even in smaller structural variants outside of chromosome 21, the putatively CHD-causative intervals within AVSD associated structural variants have proven difficult to define. A recent survey of the 3p25-ter deletion does not definitively localize the putative AVSD-specific locus to *CRELD1*, which suggests that there may be unappreciated gene regulatory elements which when halpoinsufficient confer risk for CHD, or that a *CRELD1* AVSD-specific interval is incompletely penetrant Shuib et al., 2009. Other studies of AVSD associated structural variation at the 8p23.1 locus apparently demonstrate that multiple loci within a large deletion may independently confer risk for risk for the expression of a CHD phenotype. The extension of the 8p23.1 deletion to *SOX7* within this large locus is observed to confer additional risk for different types of CHD over that of the canonical cardiac transcription factor *GATA4* Wat et al., 2009. In the context of the observations of DS, 8p23.1, and 3p25-ter, the deletions we report here are significantly smaller with fewer genes or other obvious functional elements requiring experimental validation.

We did not detect a large unique CNV on chromosome 21 for all 29 subjects with isolated sporadic AVSD. A causative role of genomic rearrangements in sporadic CHD is supported by at least one of the two CNVs we report here. Taken together, 2 of 29 (6.8%) normal patients with AVSD for whom a potential genetic etiology was previously unknown were found to have large CNVs. Recently, copy number variants (CNVs) have been implicated in the etiology of nonsyndromic CHD such as tetralogy of Fallot (ToF) Thienpont et al., 2007; Rauch et al., 2010. One study of 512 individuals with ToF excluded 22q11.2 deletion syndrome and extra-cardiac congenital defects to focus on sporadic non-syndromic disease and discovered recurrent variants at 1q21.1, 3p25.1, and 7p21.3 in 10% of enrolled cases Greenway et al., 2009. Thus, for one particular type of non-syndromic CHD a fraction is likely explained by de novo CNVs. Large sporadic CNVs are also observed in at least 3% of a mixed cohort of non-syndromic patients with isolated CHD Erdogan et al., 2008. The unique CNV hit rate of 6.8% in our normal patients with sporadic AVSDs is similar to the rates reported in those other studies of CHD. The absence of findings in at least 27 patients suggests that other categories of genetic variation that were not assessed in this study or still undiscovered environmental risk factors are responsible for the in utero development of AVSDs. Additionally we detected only one additional unique CNV in 50 patients with AVSD and DS. Future comprehensive studies will assess the complete spectrum of genetic variation including SNPs (both common variants and de novo mutations) and structural variants such as CNVs in patients with CHD.
