# Supplementary material for: Rare Copy Number Variants in Isolated Sporadic and Syndromic Atrioventricular Septal Defects
Source: Am J Med Genet A. 2012 Apr 23;158A(6):1279–84. doi: 10.1002/ajmg.a.35315 (PMC3564951; doi:10.1002/ajmg.a.35315)
Supplement: Supplementary file 1 [file ajmg158A-1279-sd1.doc]

**SUPPLEMENTARY INFORMATION**

| **Supplementary Table I** | | |
| --- | --- | --- |
|  | DS (n=50) | Euploid (n=29) |
| Male Sex | 26 (52%) | 22 (76%) |
| ECD, partial | 8 (16%) | 19 (65%) |
| ECD, complete | 42 (84%) | 10 (35%) |

| **Supplementary Table II** | | | | |
| --- | --- | --- | --- | --- |
| A. | Location of Predicted Heart Enhancers (hg18) | | |  |
|  | chr3:165653978-165654748 | |  | |
|  | chr3:165389690-165390012 | |  | |
|  | chr3:165012412-165012855 | |  | |
|  | chr3:164911475-164911890 | |  | |
|  |  |  |  | |
| B. | mIRNA | Cardiac Targets | Comments | |
|  | mIR-1263 | *KRL4* | key transcription factor regulator of pressure-induced hypertrophy and programming of iPS cell pluripotency | |
|  |  | *MID1* | microtubule associated protein causative of Opitz sydrome which often is accompanied by CHD | |
|  |  | *PDGFRB* | platelet derived growth factor receptor critical for epicardial cell migration | |
|  |  | *PPP2R2C* | regulator of intracellular cardiomyocyte calcium | |
|  |  | *CDH2* | cadherin component of cardiac desmosomes | |
|  |  | *CHD7* | ATP dependent helicase mutated in CHARGE syndrome | |
|  |  | *E2F3* | transcription factor critical in myocardial development | |
|  |  | *FGFR1* | fibroblast growth factor receptor required for cardiac development | |
|  |  | *SMAD7* | required signaling molecule for cardiac development and mutated in sporadic CHD | |
|  |  | *DICER1* | key component of post-translational RNA degradation necessary for cardiac development | |
|  |  | *DYRK1A* | cardiac expressed signalling protein on chromosome 21 | |
